# Short- to midterm outcomes following pyrocarbon glenohumeral hemiarthroplasty: a retrospective review

**DOI:** 10.1016/j.jsea.2026.100067

**Published:** 2026-07-21

**Authors:** Lindsey G. Droz, Alexandra L. Damron, Eoghan T. Hurley, Samuel G. Lorentz, Oke A. Anakwenze, Yaw D. Boachie-Adjei, Grant E. Garrigues, Christopher S. Klifto, Jonathan F. Dickens

**Affiliations:** a Department of Orthopaedic Surgery, Duke University Medical Center, Durham, NC, USA; b Department of Orthopaedic Surgery, Midwest Orthopaedics at Rush, Chicago, IL, USA

**Keywords:** Pyrocarbon, Pyrolytic carbon, Shoulder hemiarthroplasty, Outcomes, Glenohumeral osteoarthritis, Glenoid erosion, Overstuffed

## Abstract

**Background:**

Glenohumeral osteoarthritis (GHOA) in young patients remains challenging due to high revision rates following arthroplasty in active individuals. The purpose of this study was to evaluate clinical outcomes and radiographic parameters following pyrocarbon (PyC) hemiarthroplasty (HA) in patients with GHOA.

**Methods:**

A retrospective review was performed at a single academic institution from 2015 to 2025 including patients who underwent PyC HA with a minimum of one year of clinical and radiographic follow-up. Mean clinical and radiographic follow-up was 2.7 years (range, 1-7.5 years). Patient-reported outcome measures and range of motion (ROM) were recorded pre-operatively and at final follow-up. Glenoid morphology and erosion were assessed using the Walch, Sperling, and Goya classifications, along with critical shoulder angle (CSA), lateral glenohumeral offset (LGHO), humeral subluxation index, and circle method. A 2-tailed *t*-test evaluated differences between pre-operative and post-operative values with statistical significance set at *P* < .05. Normality of continuous variables was confirmed using the Mann-Whitney *U* test prior to further analysis.

**Results:**

Twenty-seven shoulders in 26 patients were included (16 males; 10 females), with a mean age of 47.4 years. The mean radiographic follow-up was 2.7 years (range, 1-7.5). Glenoid resurfacing was performed in 6 patients (23.1%). There were no fractures, dislocations, or infections. Two patients (7.6%) required revision to reverse shoulder arthroplasty at a mean of 1.3 years due to rotator cuff pathology. Twelve patients (46.2%) were overstuffed. Glenoid erosion progressed in 12 patients (46.2%), 7 of whom were overstuffed (58.3%). Among patients with erosion progression, changes in CSA and LGHO were not statistically significant. Pre-operative and post-operative subluxation was noted in 26.9% (n = 7) and 19.2% (n = 5), respectively. Significant post-operative improvements were observed in American Shoulder and Elbow Surgeons (*P* = .029) and Single Assessment Numeric Evaluation (*P* = .001) scores. ROM improved in forward flexion (*P* = .040), abduction (*P* = .006), and external rotation (*P* = .040).

**Conclusion:**

Patient satisfaction and ROM improved post-operatively following PyC HA. Revisions were related to rotator cuff pathology rather than glenoid erosion. CSA and LGHO did not significantly differ in patients with erosion progression, approximately half of whom were overstuffed. PyC HA remains a viable treatment option for GHOA in younger patients.

Glenohumeral osteoarthritis (GHOA) in the young patient represents a challenging clinical problem given the combination of high functional demands and concerns regarding implant longevity with arthroplasty.^32^ The impact on quality of life is particularly pronounced in this population who require reliable shoulder function for occupational and recreational activities with further effect on sleep quality and psychological well-being.^22^ Treatment options for GHOA in young patients begin with conservative management and joint-preserving arthroscopic procedures^3^^,^^24^; however, when arthroplasty becomes necessary, implant selection remains controversial. Anatomic total shoulder arthroplasty (TSA) provides reliable pain relief and functional improvement, with survivorship ranging from 95% to 100% at 0 to 10 years but declining to 71% to 84% at 11 to 15 years and 61% to 64% beyond 15 years in patients under 50 years.^28^ Reverse TSA has demonstrated lower short-term revision rates in young patients; however, the limited revision options necessitate careful patient selection.^16^

Traditional metal hemiarthroplasty (HA), typically utilizing cobalt-chromium bearing surfaces, was historically employed to avoid glenoid component complications. However, long-term outcomes have been disappointing. At a mean follow-up of 17.2 years, only 25% of patients reported satisfactory outcomes, with revision rates of 22.7% predominantly due to glenoid erosion.^18^^,^^31^ TSA has demonstrated significantly better range of motion (ROM), pain relief, subjective shoulder value, and Constant scores compared with metal HA, with no significant difference in longitudinal survivorship.^26^ Pyrocarbon (PyC) has emerged as an alternative bearing surface with theoretical advantages over traditional metal alloys. The material properties of PyC, including an elastic modulus similar to cortical bone and reduced wear rates in vitro, may result in less abrasion compared with metal bearing surfaces, theoretically decreasing glenoid-sided pain and erosion.^13^^,^^21^

The purpose of this study was to evaluate clinical outcomes and radiographic parameters following PyC HA in patients with GHOA. The hypothesis was that there would be significant improvement in patient-reported outcomes with minimal clinically significant glenoid erosion at final follow-up.

## Methods

### Study design

A retrospective cohort study was conducted at a single academic institution, which was determined to be exempt from institutional review board review. Patients who underwent PyC glenohumeral HA between 2015 and 2025 were identified through procedural and implant databases. All procedures were performed by fellowship-trained shoulder and sports medicine surgeons. Inclusion criteria consisted of patients undergoing primary PyC HA for treatment of primary or secondary GHOA with a minimum of one year of radiographic follow-up. Patients with prior shoulder arthroplasty were excluded. Concomitant procedures, including glenoplasty with fine burring to remove any central ridges and glenoid resurfacing with version correction, were recorded. Demographic variables, surgical indications, and perioperative details were obtained from the electronic medical record.

### Demographics

Twenty-seven shoulders in 26 patients were included (16 males; 10 females) with an average age of 47.4 years at the time of surgery (range, 26-68) and a diagnosis of primary osteoarthritis (n = 13) and secondary arthritis (n = 13) in the setting of avascular necrosis (n = 11) and post-stabilization arthropathy (n = 2). The average radiographic follow-up was 2.7 years (range, 1-7.5 years). The average body mass index was 27.8 ± 5.0 (range, 20-44), with 73% of patients denying tobacco use (n = 19). There were no patients diagnosed with diabetes, osteopenia, or osteoporosis. The majority of patients (65.4%) had concentric glenoid erosion patterns as assessed on pre-operative computed tomography with A1 (n = 11) and A2 (n = 6), with eccentric patterns comprising only 30.8% (Table I).

**Table I:** Patient demographics.

Number of shoulders	27	
Number of patients	26	
Gender	Male	16
	Female	10
Mean age at surgery	47.2 ± 10.0 (26-68)	
Body mass index	27.8 ± 5.0 (20-44)	
Tobacco use	Never	19
	Former	6
	Some days	1
Laterality	Right	15
	Left	11
Primary osteoarthritis		13
Secondary osteoarthritis	Avascular necrosis	10
	Other	3
Number of previous surgeries	Ipsilateral	5∗
Walch classification (CT)	A1	11
	A2	6
	B1	3
	B2	1
	B3	1
	C	3
	D	0
	No pre-operative CT	1

*CT*, computed tomography.

∗ Previous surgeries to include arthroscopic débridement ± subacromial decompression ± biceps tenodesis/tenotomy, removal of hardware.

### Patient-reported outcome measures

Patient-reported outcome measures included the American Shoulder and Elbow Surgeons (ASES) score^23^^,^^29^ and the Single Assessment Numeric Evaluation (SANE) score.^38^ Active ROM, to include forward elevation, external rotation at the side, and internal rotation, recorded pre-operatively and at final follow-up.

### Classification systems

Standardized true anteroposterior (Grashey) radiographs were obtained pre-operatively and at final follow-up in accordance with established imaging protocols for evaluation of GHOA.^2^ Pre-operative glenoid wear severity was graded using the Sperling classification.^30^ The Walch classification,^36^ including contemporary modifications (2016) describing advanced posterior wear patterns such as the B3 glenoid,^4^ was used throughout this article. The modified classification identifies the B3 subtype as a monoconcave glenoid with posterior humeral head subluxation, a distinction critical for implant selection and surgical planning. All patients underwent pre-operative computed tomography for glenoid characterization. Post-operative glenoid erosion progression was evaluated using both the Sperling classification^30^ and the Goya classification.^15^ The Sperling system categorizes erosion as: grade 1 (no erosion), grade 2 (subchondral erosion), grade 3 (medialization), and grade 4 (medialization to the base of the coracoid). The Goya classification further characterizes the location and pattern of erosion in the sagittal plane^15^ detailing grade 0 (no remarkable erosion), grade 1 (joint space narrowing), grade 2 (contact but no erosion), and grade 3 (a-anterior glenoid partial erosion, b-superior glenoid partial erosion, c-concentric erosion).

### Radiographic parameters

**Figure 1:**
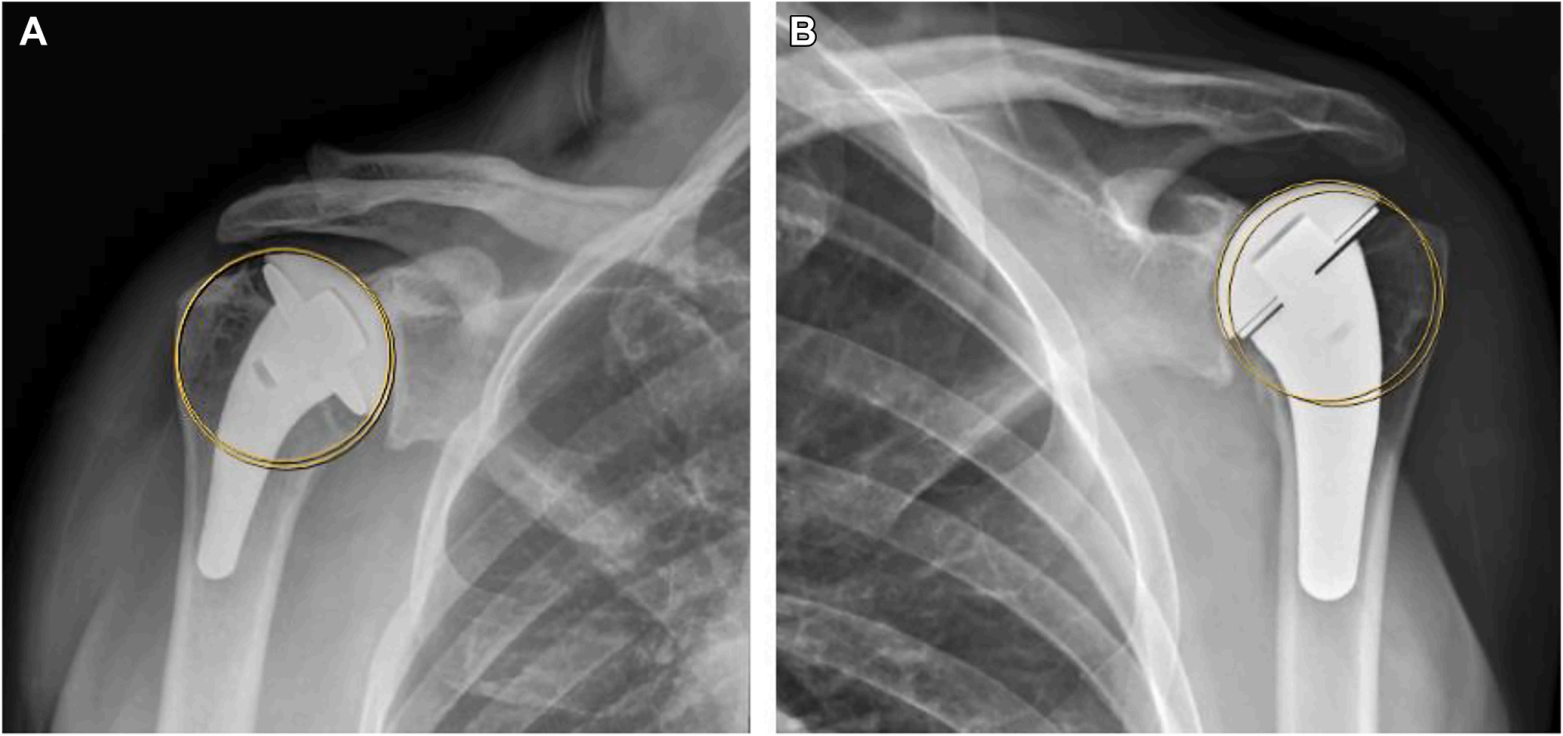
Circle method. (**A**) Anatomic reconstruction of humeral head with < 2 mm variation of the center of rotation of the native humeral head. (**B**) Nonanatomic reconstruction with 3.83 mm of overstuffing from the native humeral head to pyrocarbon implant at the glenohumeral articulation.

Radiographic parameters were measured pre-operatively and at final follow-up. The critical shoulder angle (CSA), measured on true anteroposterior radiographs as described by Moor et al,^25^ was calculated as the angle between a line connecting the superior and inferior glenoid rims and a line from the inferior glenoid rim to the lateral acromial edge. Lateral glenohumeral offset (LGHO), as previously described,^37^ was measured to quantify glenoid medialization and humeral offset. Post-operative implant sizing and anatomic reconstruction were assessed using the circle method for detection of humeral head overstuffing.^14^ Overstuffing was defined as >2 mm deviation between the native humeral head circle and the implant contour (Fig. 1). The humeral subluxation index was used to assess posterior subluxation of the humeral head relative to the glenoid. The humeral subluxation index is calculated by dividing the distance from the anterior glenoid rim to the humeral head center by the total anteroposterior glenoid width, expressed as a percentage. All radiographic measurements were performed using digital imaging software.

### Statistical analysis

Descriptive statistics were calculated for all demographic, clinical, and radiographic variables. Normality of continuous variables was confirmed using the Mann-Whitney *U* test prior to further analysis. A 2-tailed paired *t*-test was used to evaluate differences in outcome measures between pre-operative and post-operative values, with statistical significance set at *P* < .05. All analyses were performed using GraphPad (GraphPad Prism, Boston, MA, USA). Continuous variables were reported as means with standard deviations. Categorical variables were reported as frequencies and percentages.

## Results

### Complications and revisions

There were no reported fractures, lesser tuberosity nonunions, dislocations, or infections. Two revisions (7.6%) to reverse shoulder arthroplasty were performed for rotator cuff pathology (n = 2) at approximately 1.3 years post-operative. Twelve (46.2%) of the patients were overstuffed with progression of glenoid erosion in the noted 12 patients (46.2%), of which 7 (58.3%) were also overstuffed (Table II). Notably, of the 53.8% patients who were not overstuffed (14/26) with 19% of these patients showing some evidence of glenoid erosion. Neither patient that required revision had radiographic evidence of postoperative posterior humeral head subluxation (Table III).

**Table II:** Characteristics of patients with progressive glenoid erosion vs. no erosion progression.

Qualitative glenoid erosion by Sperling classification		Pre-operative		Post-operative		*P* value	Operative considerations	
Yes	46.2% (12/26)							
		LGHO	53.1 ± 6.0 (42-61)	LGHO	50.2 ± 4.6 (43-59)	.2148		
		CSA	30.7 ± 3.7 (22-38)	CSA	30.7 ± 3.8 (21-36)	.8454		
		Posterior subluxation (>55%)	25% (3/12)	Posterior subluxation (>55%)	33% (4/12)			
				Revision	0% (0/2)		Overstuffed	58.3% (7/12)
							Ream and run	25.0% (3/12)

Qualitative glenoid erosion by Sperling classification		Pre-operative		Post-operative		*P* value	Operative considerations	

No	53.8% (14/26)							
		LGHO	51.59 ± 5.88 (35-59)	LGHO	49.44 ± 3.18 (43-55)	.2397		
		CSA	30.9 ± 3.68 (27-39)	CSA	30.0 ± 4.39 (21-37)	.5617		
		Posterior subluxation (>55%)	4/14 (28.6%)	Posterior subluxation (>55%)	1/14 (7.14%)			
				Revision	100% (2/2)		Overstuffed	5/14 (35.7%)
							Ream and run	3/14 (21.4%)

*CSA*, critical shoulder angle; *LGHO*, lateral glenohumeral offset.

**Table III:** Complications and revisions.

Revision	2 (7.69%)	Revision indication	Revision procedure	Indication	Walch classification	Glenoid resurfacing	Previous operations	Overstuffed	Glenoid erosion	Pre-operative subluxation	Post-operative subluxation
		Rotator cuff pathology	Reverse total shoulder arthroplasty	Primary GHOA	C	No	0	Yes	No	Yes	No
		Rotator cuff pathology	Reverse total shoulder arthroplasty	AVN	A2	No	0	No	No	No	No

*GHOA*, glenohumeral osteoarthritis; *AVN*, avascular necrosis.

### Radiographic assessment of glenoid erosion

Glenoid resurfacing was performed in 6 patients (23.1%). Of these patients, all surgical techniques were consistent with corrective reaming of the central ridge to create a single concavity in the setting of biconcavity (B2 glenoid) or extreme retroversion (B3 glenoid). Of the patients with progression of glenoid erosion according to the Sperling and Goya classification (46.2%), the average change in pre-operative and post-operative CSA and LGHO were not statistically significant (Table IV). Five (41.7%) of the 12 patients that had some radiographic glenoid erosion progression were IIIb with superior glenoid erosion (Table IV). Notably, only 25.0% of patients (n = 3) with glenoid erosion progression underwent corrective glenoid reaming (Table II). Of the patients with glenoid erosion, pre-operative and post-operative subluxation was found in 25% (3/12) and 33.3% (4/12), respectively (Table II).

**Table IV:** Radiographic assessment of glenoid erosion and acromial morphology.

Pre-operative				Post-operative				*P* value
Qualitative glenoid erosion								
	Sperling	I II III	9 12 5		Sperling	I II III	4 10 12	___
					Goya	I II IIIa IIIb IIIC	3 13 1 5 4	___
Quantitative glenoid erosion								
	LGHO	52.0 ± 5.8 (35-61)			LGHO	49.9 ± 3.9 (43-59)		.1474
Quantitative acromial morphology								
	CSA	30.7 ± 3.6 (22-39)			CSA	30.4 ± 4.1 (21-37)		.7762

*CSA*, critical shoulder angle; *LGHO*, lateral glenohumeral offset.

### Patient-reported outcome measures and range of motion

The post-operative mean was noted to improve in comparison to pre-operative for ASES (pre-op 41.9 ± 26.7; post-op 76.1, ± 29.0, *P* = .029) and SANE (pre-op 34.0 ± 21.6; post-op 86.0 ± 15.3, *P* = .001) scores. There was a mean reported increase in ROM in pre- and post-operative comparison in forward flexion (pre-op 117.1 ± 42.3; post-op 144.7 ± 30.9, *P* = .040) abduction (pre-op 75.0 ± 26.8; post-op 113.8 ± 39.0, *P* = .006), and external rotation (pre-op 36.8 ± 22.5; post-op 51.8 ± 14.8, *P* = .040) at final follow-up (Table V).

**Table V:** Patient-reported outcome measures and range of motion.

Pre-operative			Post-operative			*P* value
	ASES	41.9 ± 26.7 (10-90)		ASES	76.1 ± 29.0 (28-100)	.029
	SANE	34.0 ± 21.6 (0-50)		SANE	86.0 ± 15.3 (50-98)	.001
	Forward flexion	117.1 ± 42.3 (40-175)		Forward flexion	144.7 ± 30.8 (90-180)	.040
	Abduction	75.0 ± 26.7 (45-140)		Abduction	113.8 ± 39.0 (60-170)	.006
	External rotation at 0°	36.8 ± 22.5 (5-70)		External rotation at 0°	51.78 ± 14.8 (20-65)	.040

*ASES*, American Shoulder and Elbow Surgeons; *SANE*, Single Assessment Numeric Evaluation.

## Discussion

### Key findings

The most important finding of this study was that patient satisfaction scores and ROM were improved throughout the duration of the study, with a low number of revision shoulder arthroplasty. The revisions that did occur were related to rotator cuff pathology rather than glenoid erosion. During the time of this implantation, the trials provided were smaller than the actual final implants and consistent with other early studies^6^ as almost half of the patients were found to have been overstuffed according to the circle method. Of the patients found to be overstuffed (58.3%), half were also noted to progress in radiographic evidence of glenoid erosion at final follow-up. There was no significant difference in the CSA and LGHO measurements in patients with glenoid erosion to serve as a predictor of rotator cuff failure or quantitative measure of glenoid erosion.

### Clinical outcome measures

Historically, traditional metal HA was deemed functionally inferior to anatomic TSA. However, recent return to play studies indicate, among PyC patients, 91% returned to work and 88% returned to sports, with overall satisfaction of 88%.^6^ Garret et al^9^ similarly demonstrated sustained clinical improvement from short-term (2-4 years) to midterm (5-9 years) follow-up, with Constant scores improving from 29.3 pre-operatively to 76.7 at 2 years and maintained at 80.8 at 6 years, while ASES scores improved from 23.7 to 87.0 and 92.3, respectively. Griswold et al^12^ reported significant improvements across all outcome measures at a mean 73-month follow-up with a 7-year revision-free survival rate of 95.7%. These findings are consistent with our cohort, which demonstrated significant improvements in ASES and SANE scores along with an increased functional ROM.

### Implant selection

While PyC HA demonstrates superior outcomes compared to metal HA, the comparison with TSA remains nuanced. The New Zealand National Joint Registry reported that patients treated with TSA had superior Oxford Shoulder Scores compared to PyC HA, with the difference exceeding the Minimal Clinically Important Difference of 4.3; however, there was no difference in revision rates between the groups.^8^ More recently, McBride et al^20^ compared PyC humeral resurfacing to best-in-class anatomic TSA prostheses from the Australian registry and found comparable survivorship at 10 years (7.7% vs. 9.0% cumulative percent revision), suggesting PyC HA may be a viable alternative in appropriately selected patients. Importantly, the predominant reason for revision differed between groups: implant breakage (35%) for resurfacing PyC vs. loosening (33.9%) for TSA.^20^ This finding has implications for patient counseling and implant selection, particularly in young, active patients where glenoid component longevity remains a concern.

### Implant survivorship

The implant survivorship of PyC HA in limited short- and midterm follow-up offers promising results. Boileau et al^5^ recently reported a 94% revision-free survival at 5 years and 89% at 10 years, with a mean glenoid erosion of only 1.4 mm at 5.5 years, primarily occurring in the first year then slowing substantially.^17^ Clinical success rates were 82.7% for PyC vs. 66.8% for cobalt-chromium (*P* < .001).^13^ From the historical literature, we hypothesized the primary cause of revision cases would remain glenoid erosion; however, in our cohort, the 2 revision cases were due to rotator cuff failure. This finding aligns with the Australian registry data demonstrating that pain, prosthesis fracture, and infection, rather than glenoid erosion, were the key reasons for revision in PyC HA.^21^ The role of rotator cuff integrity in HA outcomes is well-established with Edwards et al^7^ demonstrating that fatty degeneration of the infraspinatus and subscapularis musculature adversely affects outcomes in shoulder arthroplasty for primary osteoarthritis. Similarly, Somerson et al^35^ found that patients with intact teres minor and subscapularis tendons had significantly better prognosis for achieving clinically important improvement after HA.

### Overstuffing and technical considerations

However, the technical demand of anatomic reconstruction in PyC HA success cannot be overstated. Nonanatomic humeral reconstruction with oversizing (occurring in 24-29% of cases) significantly increases glenoid erosion risk (odds ratio, 3.46) and revision rates (25% vs. 1.3%, *P* < .001).^5^^,^^6^ The additional 1.5-mm thickness of the metal disc under the PyC head has been identified as the main reason for oversizing of the prosthetic head, as the initial trials provided were smaller than the final implants.^6^ In our cohort, 58.3% of patients were overstuffed according to the circle method using a 2 mm threshold,^1^ with half of these demonstrating progression of glenoid erosion. This rate of overstuffing is higher than previously reported and emphasizes the importance of meticulous surgical technique and utilization of the updated trials that now accurately match the final implant for intraoperative assessment of humeral head sizing. Cointat et al^6^ specifically noted that nonanatomic reconstruction (center of rotation >3 mm from anatomic center) was associated with significantly lower functional and subjective results, more complications including subscapularis insufficiency and symptomatic glenoid erosion, and higher revision risk. However, although the literature defines 2 mm-3 mm as nonanatomic reconstruction or overstuffing, Geervliet et al^10^ found a clinically significant center or rotation deviation of 5 mm correlating to the need for revision in PyC resurfacing. The variation in overstuffing threshold and the learning curve associated with this procedure may, in part, explain the higher reported rate of overstuffing in our cohort.

### Glenoid management and erosion patterns

An additional surgical consideration is the management of the native glenoid.^19^ The 2015 study by Matsen et al^19^ specifically evaluated cases of glenoid biconcavity, retroversion, and posterior humeral head displacement finding that the ream and run procedure improved humeral head centering on the glenoid from 75% posterior to 59% posterior (*P* < .001) and increased Simple Shoulder Test (SST) scores from 5 ± 3 to 10 ± 4 (*P* < .001) at a mean 3-year follow-up. However, the outcomes seem variable with Getz et al^11^ reporting the “ream and run” technique (HA with concentric glenoid reaming) to have a 25% revision rate at a mean 2.7 years in young patients with a biconcave glenoid.^19^ However, longer-term studies have demonstrated more favorable outcomes with appropriate patient selection. Somerson et al^34^ reported that the ream-and-run procedure can be effective for advanced primary GHOA, with SST scores improving from 4.9 pre-operatively to 10.3 at the latest follow-up, and significant clinical improvement observed for both A2 and B2 glenoid types.^4^ At a minimum 10-year follow-up, Sharareh et al^33^ demonstrated that 82% of ream-and-run patients achieved SST improvement above the minimally clinically important difference, with pain scores improving from 6.5 to 0.9. Importantly, Boileau et al^5^ demonstrated that “double reaming” (corrective and concentric) of the glenoid can be performed safely in biconcave erosion (type B2/B3) without adverse effects on prosthesis survival or functional results. In our cohort, glenoid resurfacing was performed in 6 patients (23.1%) with B2 or B3 glenoids only 2 (17%) of which were noted to have radiographic progression of glenoid erosion. However, progression of glenoid erosion was noted in 46.2% of patients of the total patients (12/26) with superior erosion (Goya IIIb) being the most common pattern (42% of those with erosion progression). Radiographically, 22.8% of patients in the systematic review by Park et al^27^ had evidence of glenoid erosion at minimum 2-year follow-up, though only 2.6% experienced clinically significant erosion requiring intervention. Kleim et al^17^ characterized a biphasic pattern of glenoid erosion with PyC, demonstrating 0.8 mm of erosion in the first year followed by a significantly reduced rate of 0.3 mm per year thereafter. This pattern suggests that early erosion may represent settling or adaptation rather than progressive wear, which has important implications for interpreting radiographic findings in the early post-operative period.

### Limitations

Limitations of the study included the inherent bias intrinsic to the retrospective study design and small sample size. Firstly, although the average follow-up was 2.7 years up to 7.5 years, the minimum follow-up time of one year may not capture glenoid erosion and clinical failure. These results should therefore be interpreted as short-term outcomes. The lack of a comparison group limits our ability to draw conclusions about the relative efficacy of PyC HA compared to other treatment options. Furthermore, patient-reported outcome measures were not available for all patients at all time points, which may introduce selection bias. Future studies should include multicenter, prospective comparative designs (e.g., PyC HA vs. modern anatomic TSA) with larger cohorts and minimum 5-year follow-up to better define the role of PyC HA in young patients with GHOA.

## Conclusion

At short-term follow-up (mean 2.7 years, range 1-7.5 years), patient satisfaction and ROM improved post-operatively following PyC HA. Revisions were related to rotator cuff pathology rather than glenoid erosion. CSA and LGHO did not significantly differ in patients with erosion progression, approximately half of whom were overstuffed. PyC HA remains a viable short-term treatment option for GHOA in younger patients; longer-term follow-up is needed to confirm durability of these results.

## Disclaimers

Funding: No funding was disclosed by the authors.

Conflicts of interest: Eoghan T. Hurley is a board member for Arthroscopy, European Society for Surgery of the Shoulder and Elbow, and the *Journal of Shoulder and Elbow Surgery*.

Oke Anakwenze reports professional activities at Exactech, Inc and receives royalties from SutureTech.

Yaw D. Boachie-Adjei is a paid presenter or speaker for DJ Orthopaedics and holds stock/stock options with K2M.

Grant E. Garrigues is a board member for Academic Orthopaedic Consortium, American Shoulder and Elbow Surgeons, Arthroscopy Association of North America, and the *Journal of Shoulder and Elbow Surgery*; is a paid consultant for Mitek, Restor3d, Tornier, and DJ Orthopaedics; receives royalties or other financial or material support from DJ Orthopaedics, Elsevier, and Tornier; is a paid presenter or speaker for DJ Orthopaedics; and receives stock or stock options from Aevumed, DJ Orthopaedics, Genesys, Patient IQ, Restor3d, ROM 3, Sparta Biopharma, and Zurimed.

Christopher Klifto is a board member for ASES and JAAOS and is a consultant for Smith & Nephew, Restor3d, Inc, Stryker Corporation, Smith & Nephew, and ACUMED LLC.

Jonathan F. Dickens is a board member for AAOS, American Journal of Sports Medicine, American Orthopaedic Society for Sports Medicine, American Shoulder and Elbow Surgeons, Arthroscopy Association of North America; receives research support from Arthrex, Inc, DePuy, A Johnson & Johnson Company, DoD/CDMRP, National Institutes of Health (NIAMS & NICHD), OREF, and Smith & Nephew; and holds stock or stock options with Revbio, SLACK, and Sparta Biosciences.

Any additional authors, their immediate families, and any research foundations with which they are affiliated have not received any financial payments or other benefits from any commercial entity related to the subject of this article.
